# Intentional weight loss and cancer risk

**DOI:** 10.18632/oncotarget.20671

**Published:** 2017-09-06

**Authors:** Juhua Luo, Michael Hendryx, Rowan T. Chlebowski

**Affiliations:** Juhua Luo: Department of Epidemiology and Biostatistics, School of Public Health, Indiana University Bloomington, IN, USA

**Keywords:** obesity, weight loss, intentional weight loss, cancer risk, endometrial cancer

Obesity has become a major public health issue. Currently, more than 2.1 billion people – nearly 30% of the world’s population – are either obese (BMI≥30 kg/m^2^) or overweight (25≤BMI<30 kg/m^2^). The relationships between excess body weight and cardiovascular diseases are well established [1]. Excess body weight is also recognized as an important cause for many cancers. A recent reassessment by the International Agency for Research on Cancer (IARC) based on more than 1000 epidemiologic studies identified 13 cancers with sufficient evidence to be considered obesity-related [2]. However, evaluation of body weight, cancer risk and mortality has been predominately based on increased risk associated with excess body fatness rather than reduced risks associated with weight loss. The evidence-based recommendation offered from a comprehensive review [2] was to “avoid fatness” as studies on weight loss were “sparse”. For the millions of people who are already obese, a critical question remains: can excess cancer risk and mortality from obesity be reversed through intentional weight loss? This question is even more relevant for older persons who may wonder if it is still worthwhile to lose weight at more advanced age, or whether it may be ‘too late’ to experience benefits of weight loss.

Preclinical studies [3] suggest that weight loss may reduce cancer incidence. In humans, bariatric surgery studies find 20 kg sustained weight loss associated with reduced cancer risk [4, 5]. However, clinical evidence regarding the influence of lesser weight loss achievable without surgery on cancer risk are sparse and conflicting [6, 7]. These studies are often limited by relying on self-reported weight, rather than measured weight, and by not distinguishing between intentional and unintentional weight loss. Unintentional weight loss may indicate comorbid illness and may serve to obscure the relationship between intentional weight loss and health benefit. Mixed findings from prior studies preclude a strong public health message that people who are overweight or obese could reduce their cancer risk and mortality risk by losing weight.

We reported results from a large prospective study that included measured weight and weight loss by intentionality among postmenopausal women [8]. Our study observed that compared to women with stable weight (change within ±5%), women with weight loss (≥5%) had significantly lower endometrial cancer risk (HR = 0.71, 95% CI: 0.54-0.95). The association was strongest among obese women with intentional weight loss (HR = 0.44, 95% CI: 0.25-0.78) [8]. The results for endometrial cancer provide strong evidence supporting the hypothesis that intentional weight loss among obese postmenopausal women will lower their cancer risk. However, more studies are needed to determine whether our findings are generalizable to other populations or other obesity-related cancer types. Given that sustained weight loss is difficult to maintain, it is also important to investigate whether there is benefit from short-term weight loss with the smaller degree of weight loss achievable without surgery versus sustained weight loss, and whether the benefits of weight loss are offset by any subsequent regain. Furthermore, studies of the relationship between weight change trajectories over the adult life-course and cancer and mortality risk will provide more insights into longer term risks and potential adverse consequences from weight cycling. Findings from these studies will inform an important translational public health message on how weight loss among obese people is associated with their health outcomes. Based on current findings, however, we can offer a positive clinical message that it is beneficial for obese older women to lose weight to reduce endometrial cancer risk.
